# The impact of glucose exposure on bioenergetics and function in a cultured endothelial cell model and the implications for cardiovascular health in diabetes

**DOI:** 10.1038/s41598-020-76505-4

**Published:** 2020-11-11

**Authors:** Maria Luisa Fiorello, Andrew T. Treweeke, David P. Macfarlane, Ian L. Megson

**Affiliations:** 1 Division of Biomedical Sciences, Centre for Health Science, University of the Highlands and Islands, Inverness, IV2 4JH UK; 2 Department of Diabetes, NHS Highland, Raigmore Hospital, Inverness, UK

**Keywords:** Biochemistry, Biological techniques, Cell biology

## Abstract

Cardiovascular disease is the primary driver of morbidity and mortality associated with diabetes. Hyperglycaemia is implicated in driving endothelial dysfunction that might underpin the link between diabetes and cardiovascular disease. This study was designed to determine the impact of chronic preconditioning of cells to hyperglycaemia and transient switching of cultured endothelial cells between hyper- and normo-glycaemic conditions on bioenergetic and functional parameters. Immortalised EA.hy926 endothelial cells were cultured through multiple passages under normoglycaemic (5.5 mM) or hyperglycaemic (25 mM) conditions. Cells were subsequently subjected (48 h) to continued normo- or hyperglycaemic exposure, or were switched to the alternative glycaemic condition, or to an intermediate glucose concentration (12.5 mM) and metabolic activity, together with key markers of function were measured. Cells habituated to hyperglycaemia were energetically quiescent. Functional activity, characterised by the measurement of nitric oxide, endothelin-1, tissue plasminogen activator and plasminogen activator inhibitor-1, was depressed by exposure to high glucose, with the reduction in nitric oxide production being the most notable. Function was more responsive to acute changes in extracellular glucose than were bioenergetic changes. We conclude that glucose is a key determinant of endothelial function. The study highlights the importance of chronic glucose exposure on cell phenotype and emphasises the need to pay close attention to glucose preconditioning in interpreting results under culture conditions.

## Introduction

Cardiovascular disease (CVD) is the major cause of morbidity and mortality in individuals with diabetes. Whilst Type 1 and Type 2 Diabetes Mellitus are pathologically distinct diseases, hyperglycaemia is common to both and is considered to be the cornerstone of diabetic complications, including vascular disease.

Chronic hyperglycaemia is known to induce cellular metabolic adaptation or changes in endothelial cell functionality. This phenomenon is described by some as metabolic memory^1–4^, a term first used to describe the impact of early glycaemic control on the reversibility or progression of complications associated with diabetes in human^5^ and animal^6–9^ studies.

A wide range of mechanisms have been proposed to explain hyperglycaemia-driven cellular dysfunction, including oxidative stress, advanced glycation end product (AGE) formation, epigenetic modifications and inflammation^1–4,10–13^. Central to many of these processes is the impact of glucose on cellular respiration, either directly on account of carbohydrate overload or indirectly through epigenetic modifications in mitochondrial and nuclear DNA and/or post-translational modifications of metabolic enzymes.

Changes to the function of endothelial cells induced by hyperglycaemia are also recognised in a number of studies^14–24^, but the relationship to bioenergetic modulation is poorly defined. Nitric oxide (NO) is one of the key players in endothelial function and its protective qualities are well-known to be impacted upon by several key processes thought to be related to hyperglycaemic challenge, including inflammation, oxidative stress and cofactor availability (e.g. tetrahydrobiopterin, nicotinamide adenine dinucleotide phosphate (NAD(P)H))^25^. However, NO is only one of a number of important modulators of both vasomotion, thrombosis and fibrinolysis, some of which have received much less attention in this respect.

The need for chronic exposure of cells to drive some of these processes presents a problem in traditional cell culture experiments because standard procedure typically involves cells grown in hyperglycaemic conditions (25 mM); treatments are often fairly brief and really represent deprivation of glucose from cells habituated to hyperglycaemia^21,26–30^. In order to gain a more informative picture of the impact of hyperglycaemia on cell function, cell culture models need to be considered afresh, with cells grown under normo-glycaemic conditions through multiple passages to habituate them to glucose at concentrations similar to those found in vivo.

The aim of this study was to test the hypothesis that chronic hyperglycaemia drives important changes in metabolism, redox balance and function in endothelial cells. A supplementary hypothesis was that the key changes to cell function were resistant to reversal by acute glucose normalisation.

## Results

### Preconditioning in high and normal glucose does not alter cell growth/death parameters

Cells preconditioned in high glucose (HG) and normal glucose (NG) conditions through many (n > 4) passages were indistinguishable under light microscopy (Supplementary Fig. S1A online) and grew at similar rates (Supplementary Fig. S1B online). The lack of impact of glucose concentration on these base parameters is vital knowledge prior to quantifying and interpreting subsequent experimental data.

### Effect of glucose concentration on bioenergetic parameters

**Figure 1:**
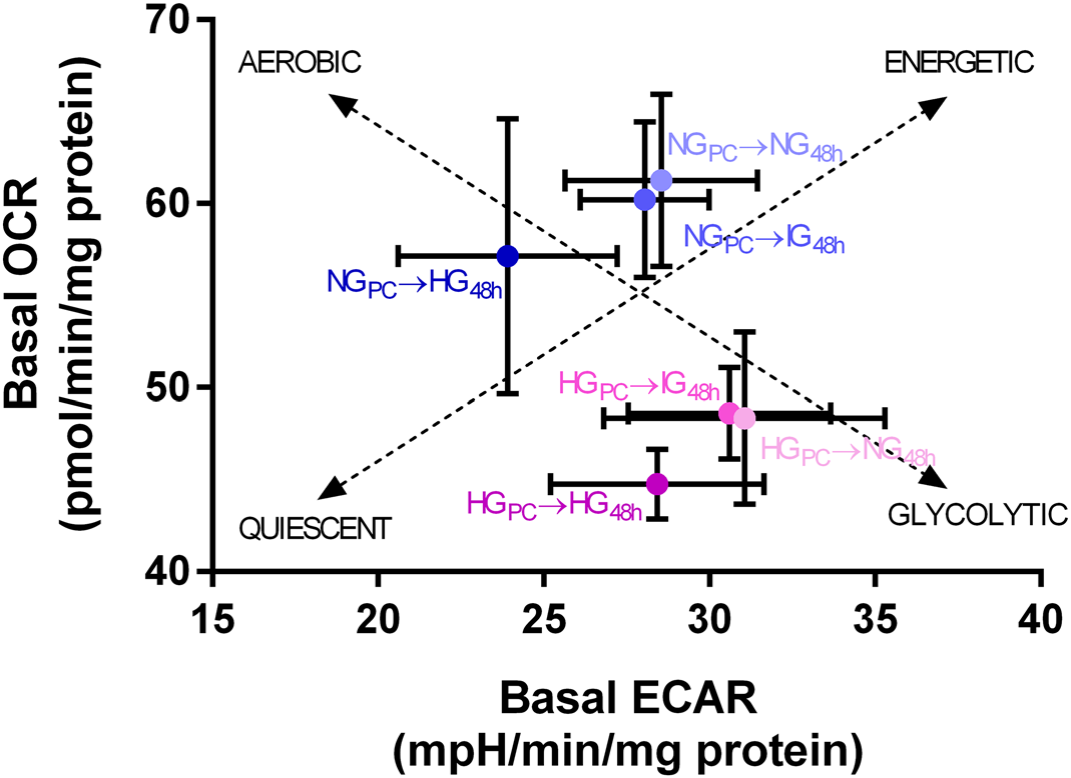
Energy map for cells cultured under various glucose conditions. HG_PC_ cells exhibit a more quiescent phenotype than NG_PC_ cells. 48 h exposure of HG_PC_ cells to lower glucose concentrations (IG or NG) drove a partial reversal of the effect, just as 48 h exposure of NG_PC_ cells with IG or HG medium partially induced the quiescent phenotype. Dotted lines represent the aerobic-glycolytic axis and the quiescent-energetic axis. Data are expressed as mean ± SEM (n = 10–12 for each mean depicted). Raw data were analysed using Seahorse Wave Software (version 2.3.0.19) prior to graphical presentation using Graphpad Prism version 6.00.

HG preconditioning (HG_PC_) and subsequent continued exposure to 25 mM glucose for a further 48 h (HG_PC_ → HG_48h_) generated a more quiescent phenotype compared to the NG control (NG_PC_ → NG_48h_), represented as a vertical reduction on the energy map (Fig. 1). The impact of exposing NG_PC_ cells to higher glucose was to instigate a slide down the energetic/quiescent axis, whereas reducing glucose in HG_PC_ cells caused a transition in the opposite direction, towards a more energetic state. There was no detectable impact of changing glucose concentrations on the aerobic-glycolytic axis of the energy map.

#### Complex V activity, glucose depletion from the medium, and maximum and spare capacity

**Figure 2:**
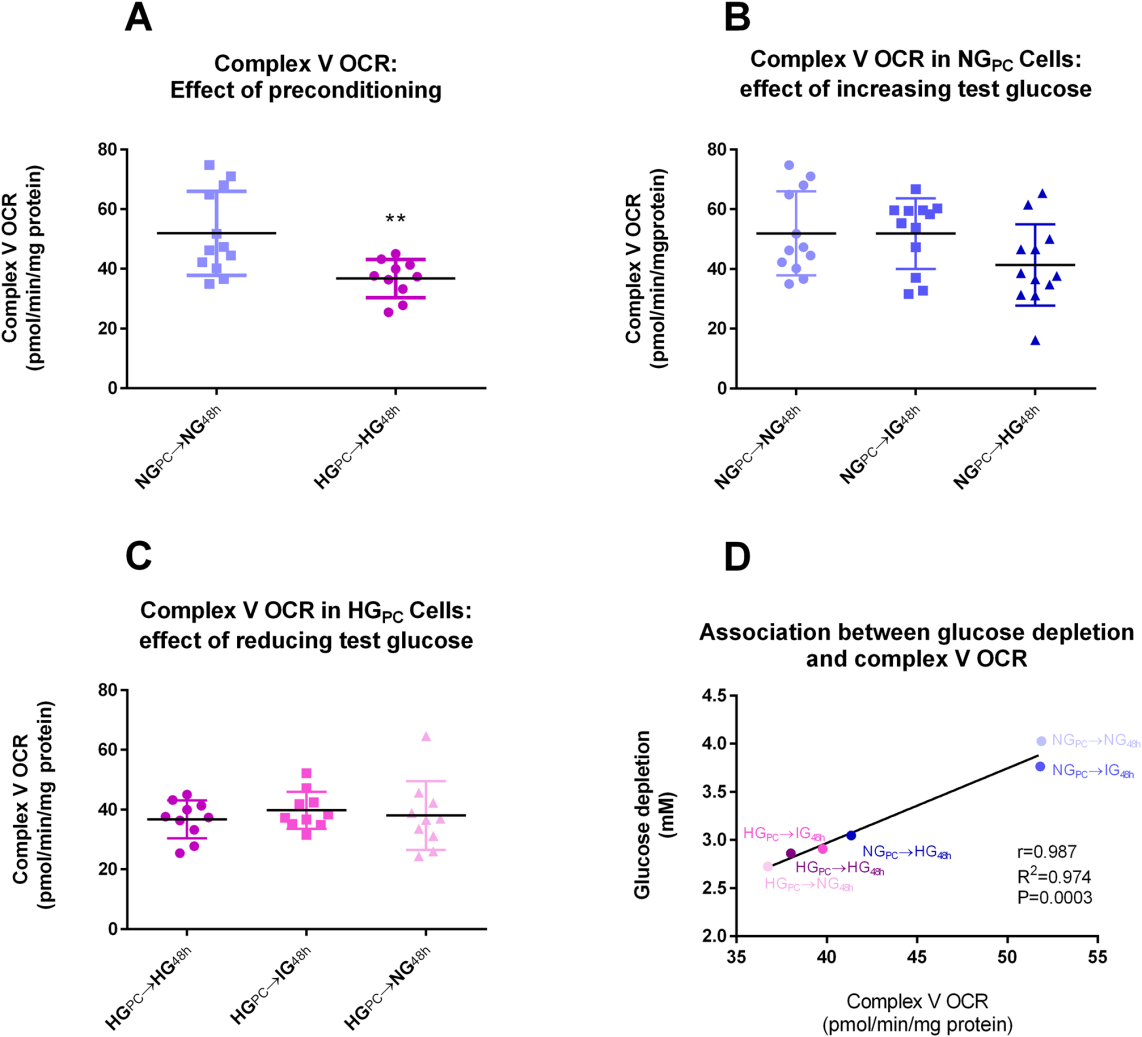
(**A**) Effect of glucose preconditioning on complex V-associated OCR (**P < 0.01; Mann Whitney U test). (**B**) Effect of 48 h exposure of increasing glucose concentrations to NG_PC_ cells (P > 0.05; Kruskal–Wallis). (**C**) Effect of 48 h exposure of decreasing glucose concentrations in HG_PC_ cells (P > 0.05; Kruskal–Wallis). (**D**) Correlation of mean complex V-associated OCR with mean glucose depletion from medium under each HG and NG condition. Raw data for glucose depletion are found in Supplementary Fig. S3. Raw data were analysed using Seahorse Wave Software (version 2.3.0.19) prior to graphical presentation using Graphpad Prism version 6.00.

Oxygen consumption rate (OCR) related to complex V activity was significantly higher (+ 45%) in NG_PC_ cells than in HG_PC_ cells (Fig. 2A) and there was a trend towards a reduction in complex V-mediated OCR in NG_PC_ cells upon 48 h exposure to increased glucose concentrations (Fig. 2B). Complex V-mediated OCR was unaffected by normalisation of glucose in HG_PC_ cells (Fig. 2C). The variation in complex V-mediated OCR across different treatments was directly correlated to glucose depletion from the medium, most likely entirely due to uptake by cells (Fig. 2D).

The original data relating to glucose depletion from media is found in Supplementary Fig. S2 online; glucose depletion was significantly lower in HG_PC_ cells, despite the theoretical concentration gradient being substantially higher (Supplementary Fig. S2A online). The effect was not induced by acute exposure (Supplementary Fig. S2B online) and not reversed by 48 h incubation with lower glucose concentrations (Supplementary Fig. S2C online).

Maximum capacity (the maximum OCR obtained following FCCP injection) and derived spare capacity (maximum OCR minus basal OCR) were both significantly lower in HG_PC_ cells (Supplementary Fig. S3A, D online). The effect was not induced by 48 h exposure of NG_PC_ cells to higher glucose concentrations (Supplementary Fig. S3B,E online) and was not reversed by exposure of HG_PC_ cells to lower glucose concentrations (Supplementary Fig. S3C,F online).

### Effect of glucose on measures of oxidative stress-associated parameters

**Figure 3:**
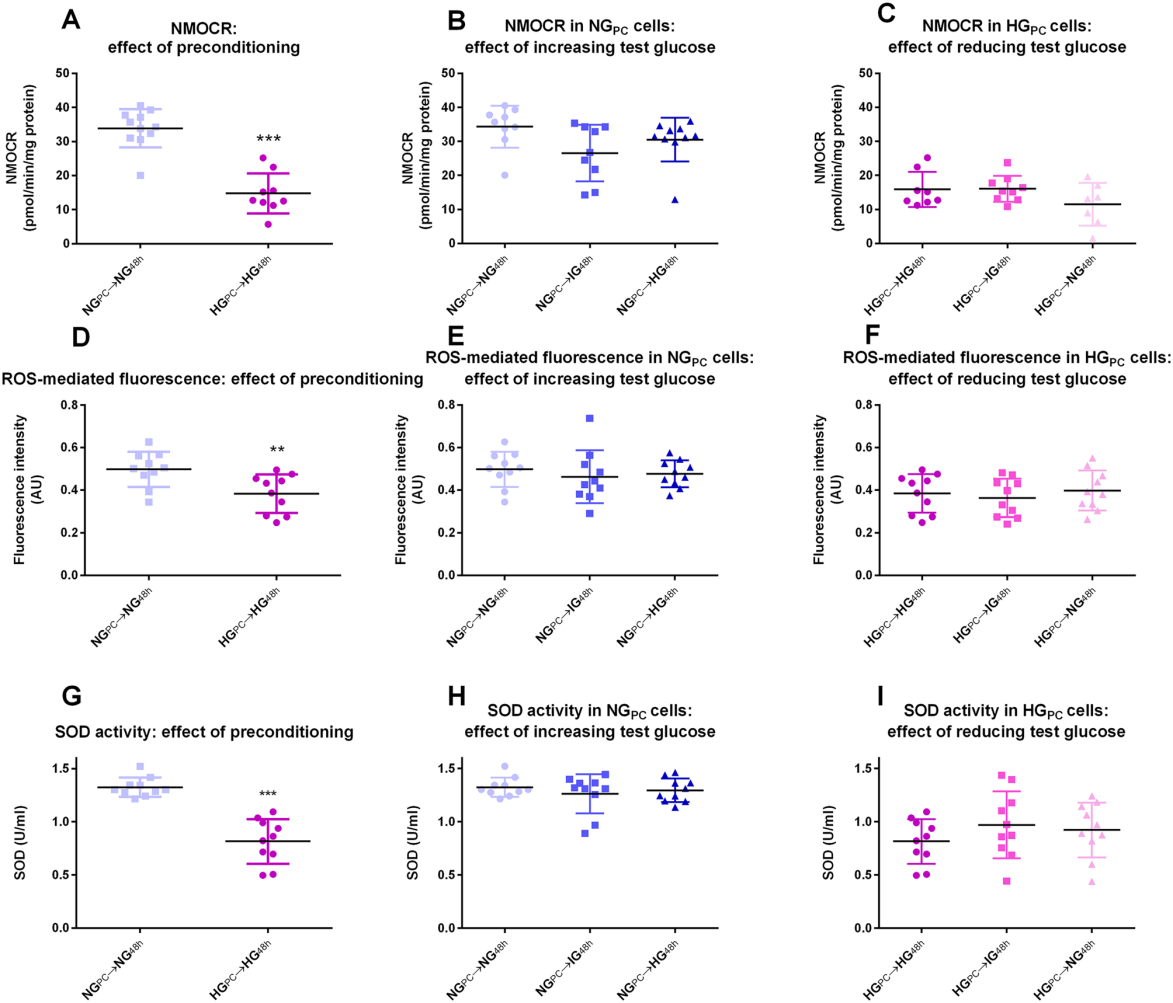
(**A**) Effect of glucose preconditioning on NMOCR (***P < 0.001; Student’s *t*-test). (**B**) Effect of 48 h exposure of increasing glucose concentrations to NG_PC_ cells (P > 0.05; One-way ANOVA). (**C**) Effect of 48 h exposure of decreasing glucose concentrations in HG_PC_ cells (P > 0.05; One-factor ANOVA). (**D**) Effect of glucose preconditioning on DFDCA fluorescence (**P < 0.01; Student’s t-test). (**E**) Effect of 48 h exposure of increasing glucose concentrations in NG_PC_ cells (P > 0.05; one-factor ANOVA). (**F**) Effect of 48 h exposure of decreasing glucose concentrations in HG_PC_ cells (P > 0.05; one-factor ANOVA). (**G**) Effect of glucose preconditioning on SOD activity (***P < 0.001; Mann Whitney U test). (**H**) Effect of 48 h exposure of increasing glucose concentrations on SOD activity in NG_PC_ cells (P > 0.05; one-factor ANOVA). (**I**) Effect of 48 h exposure of decreasing glucose concentrations on SOD activity in HG_PC_ cells (P > 0.05; one-factor ANOVA). Raw data were analysed using Seahorse Wave Software (version 2.3.0.19) prior to graphical presentation using Graphpad Prism version 6.00.

Both non-mitochondrial OCR (NMOCR; − 58%) and ROS-induced fluorescence (an indicator of total ROS/RNS; − 21%) were significantly depressed in HG_PC_ cells compared to NG_PC_ cells (Fig. 3A,D). The effect of HG_PC_ on NMOCR and ROS detection was not reversed by exposure to lower glucose concentrations for 48 h (Fig. 3B,E) and was not induced in NG_PC_ cells by exposure to higher concentrations for 48 h (Fig. 3C,F).

SOD activity was substantially reduced in HG_PC_ cells (− 38%; Fig. 3G; there was no significant impact of switching NG_PC_ cells to IG or HG media for 48 h (Fig. 3H) or of switching HG_PC_ cells to lower glucose concentrations for 48 h (Fig. 3I).

### Effect of glucose concentration on endothelial cell function

#### Nitrite generation

**Figure 4:**
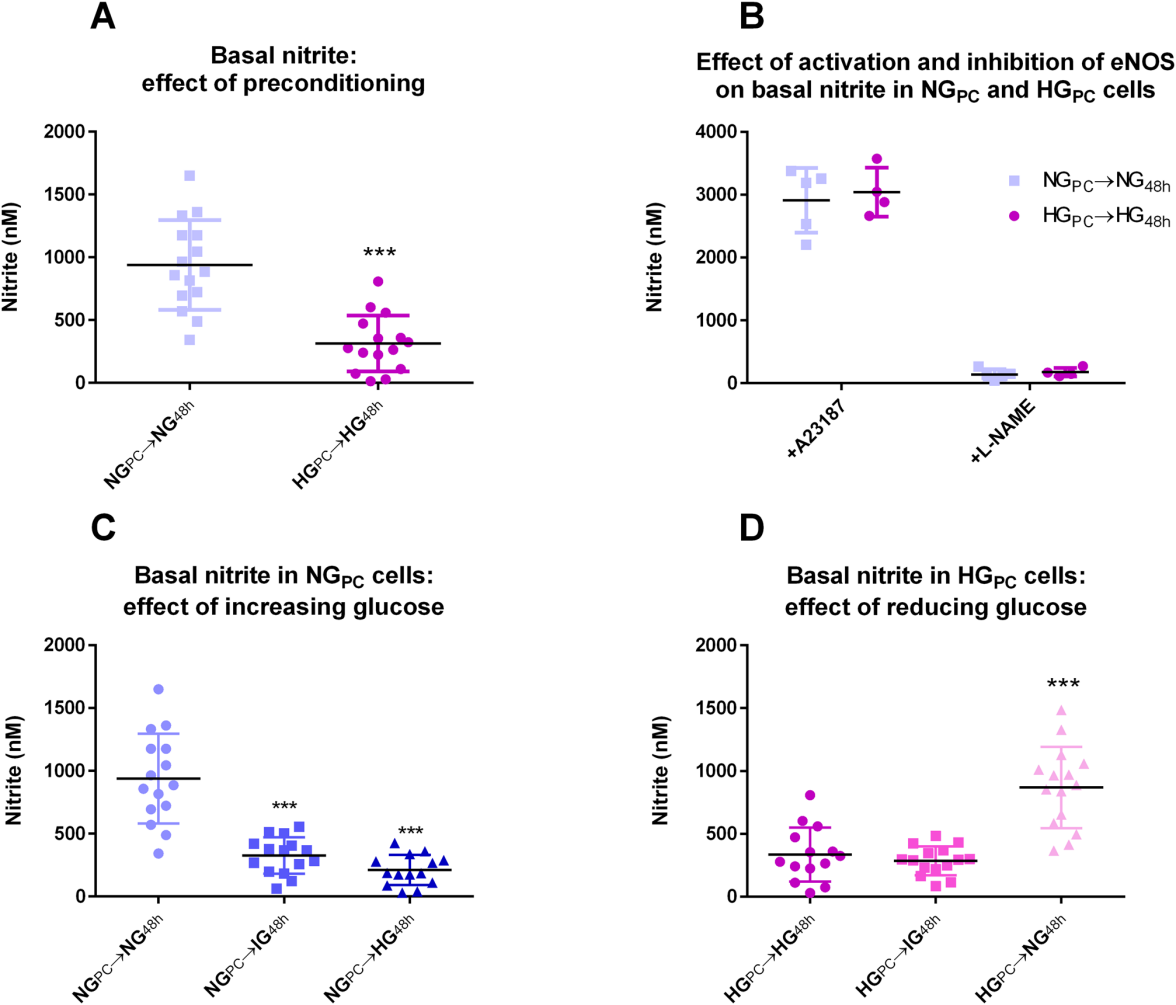
(**A**) Effect of glucose preconditioning on nitrite accumulation (***P < 0.001; Mann Whitney U test). (**B**) Effect of activation (A23187 1 μM, 1 min calcium ionophore) and inhibition (l-NAME 100 μM, 24 h) on nitrite accumulation in medium) in NG_PC_ and HG_PC_ cells (P > 0.05; Student’s *t*-tests between glucose conditions). (**C**) Effect of 48 h exposure of increasing glucose concentrations to NG_PC_ cells on nitrite (***P < 0.001; Kruskal–Wallis test with Dunn’s post-test). (**D**) Effect of 48 h exposure of decreasing glucose concentrations in HG_PC_ cells on nitrite (***P < 0.001; Kruskal–Wallis test with Dunn’s post-test). Data were analysed using Liquid Software (version 2) and presented using Graphpad Prism version 6.00 Software.

Nitrite was measured in the medium as a surrogate marker of basal nitric oxide (NO) generation. Accumulation of basal nitrite was significantly inhibited in HG_PC_ cells (Fig. 4A; − 67%), but the maximum eNOS-derived nitrite generated in cells treated with the calcium ionophore, A23187 was not affected (Fig. 4B). The source of nitrite was confirmed to be NOS on account of the substantial inhibitory effect of the NOS inhibitor, l-NAME (Fig. 4B). Accumulation of basal nitrite was significantly inhibited by 48 h incubation of NG_PC_ cells with higher glucose concentrations (Fig. 4C). Conversely, basal nitrite was increased in HG_PC_ cells exposed to NG conditions for 48 h (Fig. 4D).

#### ET-1 secretion

**Figure 5:**
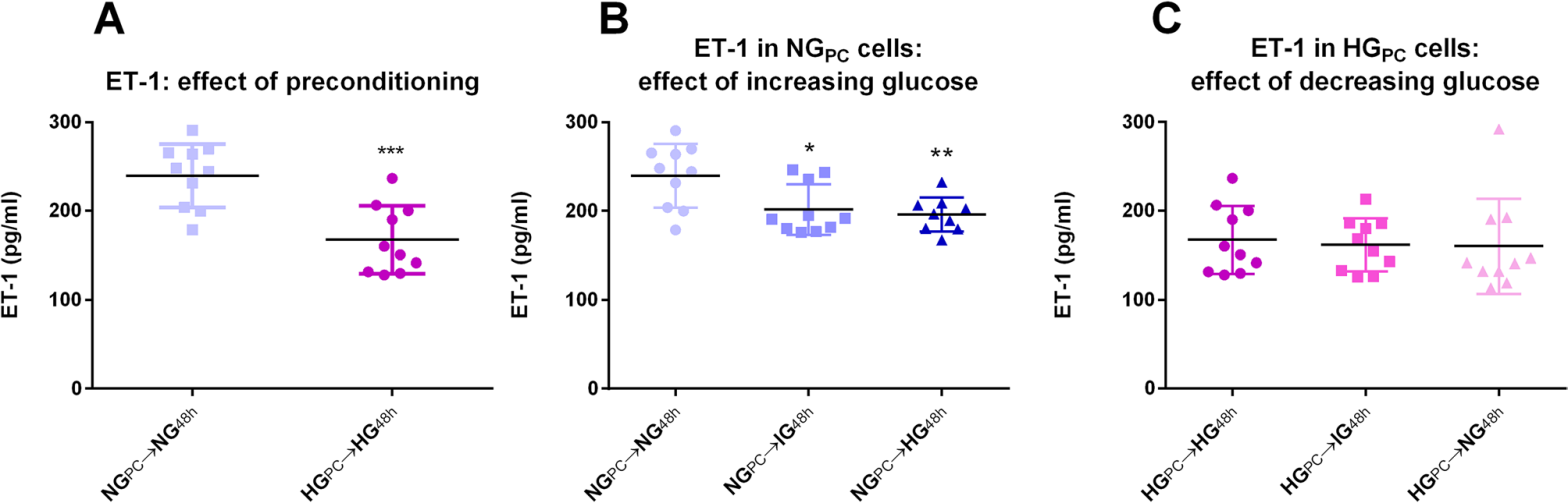
(**A**) Effect of glucose preconditioning on ET-1 (***P < 0.001; Student’s t-test). (**B**) Effect of 48 h exposure of increasing glucose concentrations to NG_PC_ cells on ET-1 (*P < 0.05, **P < 0.01; One-factor ANOVA with Bonferroni post-test). (**C**) Effect of 48 h exposure of decreasing glucose concentrations in HG_PC_ cells on ET-1 (P > 0.05; One-factor ANOVA). Data were collected using SkanIt Software 2.4.5 and further analysed and presented using Graphpad Prism version 6.00 Software.

ET-1 secretion was modestly inhibited (− 20%) in HG_PC_ cells compared to NG_PC_ cells (Fig. 5A). A trend towards this effect was seen when NG_PC_ cells were switched to IG and HG conditions for 48 h (Fig. 5B), but HG_PC_ cells were recalcitrant to any effect of NG in this regard (Fig. 5C).

#### t-PA and PAI-1 antigen

**Figure 6:**
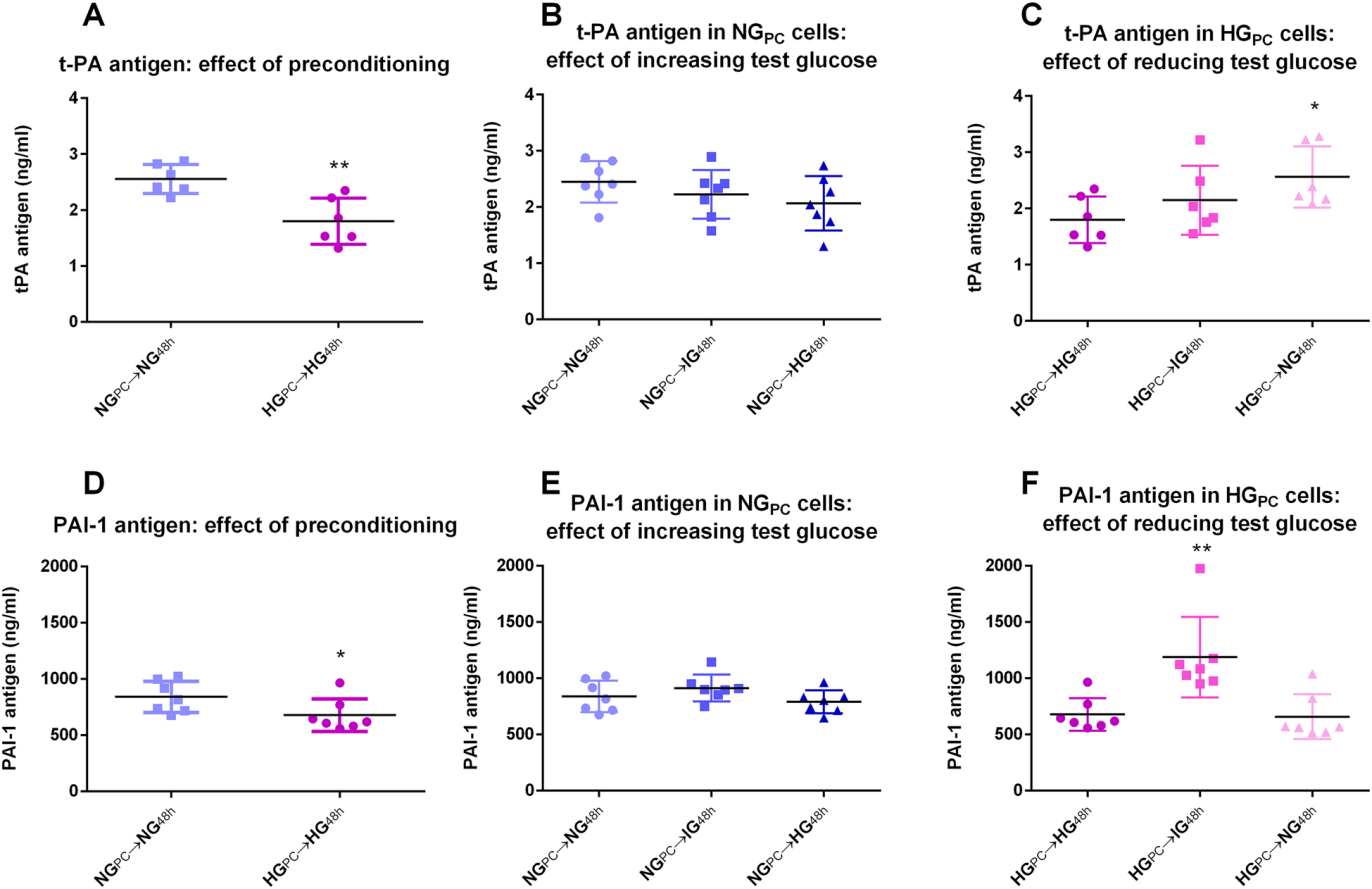
(**A**) Effect of glucose preconditioning on t-PA antigen (**P < 0.001; Student’s t-test). (**B**) Effect of 48 h exposure of increasing glucose concentrations to NG_PC_ cells on t-PA antigen (P > 0.05; One-factor ANOVA). (**C**) Effect of 48 h exposure of decreasing glucose concentrations in HG_PC_ cells on t-PA antigen (*P < 0.05; One-factor ANOVA with Bonferroni post-test). (**D**) Effect of glucose preconditioning on PAI-1 antigen (**P < 0.001; Student’s t-test). (**E**) Effect of 48 h exposure of increasing glucose concentrations to NG_PC_ cells on PAI-1 antigen (P > 0.05; One-factor ANOVA). (**F**) Effect of 48 h exposure of decreasing glucose concentrations in HG_PC_ cells on PAI-1 antigen (*P < 0.05, **P < 0.01; One-factor ANOVA with Bonferroni post-test). Data were collected using SkanIt Software 2.4.5 and further analysed and presented using Graphpad Prism version 6.00 Software.

t-PA antigen secretion into the medium was significantly reduced (-28%) under basal conditions in HG_PC_ cells compared to the NG equivalents (Fig. 6A). Treatment of NG_PC_ cells with higher glucose for 48 h showed a trend towards inhibition (Fig. 6B), while the trend was reversed in HG_PC_ cells treated with reduced glucose, reaching significance in those treated with NG (Fig. 6C).

PAI-1 was depressed (− 20%) in HG_PC_ cells compared to NG_PC_ cells (Fig. 6D). The effect was not recapitulated in NG_PC_ cells exposed to higher glucose conditions for 48 h (Fig. 6E), nor was the reverse seen in HG_PC_ cells exposed to IG or NG (Fig. 6F). Interestingly, a reduction of glucose to 12.5 mM in HG_PC_ cells resulted in a significant increase in PAI-1 (Fig. 6F). Taken in conjunction with the t-PA results, while both antigens are reduced in hyperglycaemia, the impact is marginally greater on t-PA. Given that the net fibrinolytic activity of t-PA is modulated by PAI-1 through 1:1 binding, it is reasonable to deduce that the overall effect of hyperglycaemia is a modest shift away from fibrinolytic capacity. The effect is particularly clear in cells exposed to intermediate glucose after hyperglycaemic preconditioning (Fig. 6F).

## Discussion

The data presented demonstrate that an immortalised endothelial cell line habituated to hyperglycaemic conditions through many passages display a bioenergetically and functionally distinct phenotype from cells habituated to normoglycaemic conditions. Cells habituated to hyperglycaemic conditions are substantially less metabolically active than normoglycaemic counterparts, an effect that is not replicated with acute (48 h) treatment of normoglycaemic cells with intermediate (12.5 mM) or high (25 mM) glucose. Likewise, 48 h treatment of hyperglycaemic cells with lower (intermediate or normal) glucose conditions fails to fully reverse the effect. The bioenergetic changes in hyperglycaemia (HG_PC_ cells) are associated with reduced glucose depletion from the medium in HG_PC_ cells, a depression of ROS generation and SOD activity. Several key endothelium-derived factors were assessed and all were reduced under hyperglycaemic conditions, with NO showing the most marked depression. Unlike the bioenergetic parameters, functional changes were found to bear a more dynamic association with glucose concentration; 48 h treatments were sufficient to initiate at least partial switching between the quiescent HG state and the more active NG state; this effect was bi-directional in the case of NO and ET-1. The different timescales involved for glucose-induced modulation of function, compared to bioenergetic changes, might suggest that there is no causal association between function and metabolism. However, it cannot be excluded that extended glucose exposure (in this specific case, greater than 48 h) might also alter cell bioenergetics as well as cell function over the longer time scale.

### Bioenergetics

The overwhelming impact of chronic preconditioning of EA.hy926 endothelial cells in HG media is to drive them towards a more quiescent phenotype; the glycolysis element of the effect is not induced in NG_PC_ cells following 48 h exposure to HG, but quiescence is partially induced with this acute exposure. Maximal and spare capacity, markers of metabolic flexibility, were similarly depressed by HG_PC_ but not 48 h exposure and were not reversed by acute normalization of glucose in the medium. The temporal aspect of these effects is potentially important: glycolytic alteration is clearly much slower to develop and aligns more closely with the counter-intuitive reduction in glucose uptake in HG_PC_ cells. The reason behind the substantial reduction in glucose uptake is not known, but likely involves a range of counter-regulatory measures within the cell in an effort to prevent glucose overload. Switching to energetically less efficient glycolysis might be part of this strategy, which would accelerate utilization of excess glucose and contribute to the observed reduction in maximum and spare capacity, which also are not seen with acute exposure to HG. By contrast, the migration of cells along the energetic/quiescent axis is a far more dynamic effect that is partially induced and reversed in the 48 h treatment experiments.

### Oxidative stress

The results obtained for measures associated with oxidative stress largely run counter to previous reports. NMOCR is an indicator of oxygen consumption related to all cellular oxygen consumption that persists after respiration is abolished by complex I and 3 inhibition. The sources of NMOCR are multiple, including various enzymes (NAD(P)H oxidases, xanthine oxidase, cyclo-oxygenases)^31–34^. The observed substantial and persistent reduction of NMOCR in HG_PC_ cells could be interpreted as a depression of oxidative stress in these cells, which is supported by the fluorescence measure of total ROS production and perhaps partially explained by the reduction in SOD activity. However, we cannot rule out a downregulation of various oxidases and peroxidases under HG conditions, which would resonate with the overall reduction of metabolic activity seen. What is clear is that these results are at odds with published results, that indicate hyperglycaemic exposure as the stressor that leads to COX-2 upregulation^33,35,36^ or NOX^37–39^. The precise reason for this difference is unclear at this stage, but could be due to the preconditioning element of our experiments, which is unusual compared to much of the literature, but in our view more appropriate. In addition, it is worth noting that the glucose concentration in the medium of NG-treated cells fell to what might be considered hypoglycaemic levels over 24 h, perhaps inducing glycaemic stress of a different kind.

### Function

While many of the effects of HG in terms of bioenergetics and oxidative stress are slow to develop and irreversible with acute normalisation of glucose, the impacts on key endothelial cell functions are altogether more dynamic, perhaps reflecting transient changes to physiological needs. Of the measures we undertook in this study, nitrite (a surrogate marker of NO generation) experienced the most profound effect, with ~ 70% reduction seen with both acute (48 h) and preconditioning HG treatments. eNOS regulation occurs at a range of levels, from gene expression (e.g. via the diacylglycerol protein kinase C (DAG-PKC) pathway), through substrate (l-arginine) and cofactor (NADPH, tetrahydrobiopterin) availability and phosphorylation^40–45^. The data we obtained using A23187 as a maximal activator of eNOS suggests that, given sufficient stimulus, HG cells were still fully capable of generating the same amount of NO, irrespective of glucose conditions. Equally, NOS was found to be the sole source of the NO measured via the surrogate nitrite; taken together, it seems unlikely that eNOS expression was substantially altered by glucose, but rather that the impact is downstream of synthesis. NO activity is influenced by interaction with off-target molecules, including superoxide^40,46–49^, but our data would suggest that increased interaction with superoxide under HG conditions is unlikely, given the reduced oxidative stress in these cells. Direct inhibition by glucose^50^, on the other hand, cannot be excluded. What is certain, however, is that a similar effect driven by hyperglycaemia in vivo would constitute endothelial dysfunction and contribute to a pro-atherogenic and pro-thrombotic state.

There was a HG-induced reduction in the vasoconstrictor and pro-mitogenic mediator ET-1 and the fibrinolytic modulators t-PA and PAI-1. That all three proteins are down-regulated, taken together with the apparent depression of SOD, chimes with an overall dampening of cellular activity in line with reduced metabolism. While it is not possible to reach a conclusion as to the net effect of NO/ET-1 changes on constriction and atherogenic processes, the greater impact of HG on t-PA compared to PAI-1 is likely to result in a net reduction in fibrinolytic activity, given that PAI-1 binds to t-PA in a 1:1 stoichiometry to inactivate it. Should such an effect be replicated in vivo, there would be a reduced capability to eliminate potentially harmful micro-thrombi.

### Limitations

Accurate mimicking of in vivo glycaemic exposure is all but impossible in cell culture format. While we often consider in vitro glucose treatments to be “constant”, in truth they will gradually decline as glucose is utilised. Our experimental measurements confirmed, for example, that while our NG treatment regimen was set up at 5.5 mM, the glucose concentration remaining after 24 h was often below concentrations that might be considered to be hypoglycaemic from a clinical standpoint.

It could be argued that primary human endothelial cells would have been a more appropriate cell model to use, rather than the immortalised endothelial cell line studied. However, the preconditioning stage requires numerous cell passages and primary endothelial cells are known to lose their endothelial cell characteristics over repeated passages. This would have proved to be a profound limitation to this cell model so, on balance, the EA.hy926 cell line was considered a more suitable option than primary human endothelial cells.

Detection of ROS is fraught with difficulties and no method should be considered perfect. We chose to use a fluorescent marker for ROS, which is fairly unspecific in terms of establishing the nature of the ROS generated or their source. However, the positive control used (pyocyanin) generated a substantial signal, suggesting that the probe is at least capable of detecting ROS related to mitochondrial dysfunction.

## Conclusions

Pre-conditioning cultured cells with HG through multiple passages has a profound effect on bio-energetics, antioxidant adaptation and cell function. Collectively, the effect of HG_PC_ is to establish a quiescent phenotype; the impact on bioenergetics is not replicated with relatively acute (48 h) exposure to HG and is not reversed by acute normalisation of glucose in the media. This has important implications for interpretation of results from studies conducted using standard HG_PC_ protocols—it is important to recognise that cells cultured under these conditions are likely to have assumed a different energetic phenotype to those in vivo under homeostatic control. Even the unusually high glucose conditions experienced in the diabetic state are not reflected by sustained exposure to glucose > 20 mM, as found in standard cell culture. Traditional cell culture conditions could, therefore, generate a cell phenotype that is a mis-representation of the diabetic state.

The impact on endothelial cell function is more dynamic. The detrimental effect on eNOS-derived NO is particularly profound, with important implications in terms of potential for increased vasoconstriction, mitogenesis and risk of thrombosis.

## Methods

### Materials

Unless stated otherwise, all chemicals were purchased from Sigma Aldrich (Dorset, UK). Cell culture consumables were purchased from VWR (Lutterworth, UK).

### Cell culture and growth curves

The endothelial cell line EA.hy926 (ATCC CRL-2922) retains an endothelial phenotype over repeated passages and was used in all cellular experiments. The EA.hy926 cells were cultured in Dulbecco’s Modified Eagle’s Medium (DMEM; HyClone) containing glucose at either 5.5 mM (normal glucose; NG) or 25 mM (high glucose; HG), in standard conditions of a humidified atmosphere at 37 °C and 5% CO_2_. Both media were supplemented with 1 mM sodium pyruvate, 2 mM l-glutamine, 5% penicillin/streptomycin (10,000 Units/ml penicillin and 10 mg/ml streptomycin) and 10% fetal bovine serum. Stock NG and HG cells at 90% confluence had been passaged by trypsinization (0.05% trypsin-ethylenediaminetetraacetic acid (EDTA) solution; Gibco, Thermo-Fisher Scientific), re-seeded and pre-conditioned (_PC_) in their respective media a minimum of five times to generate two separate EA.hy926 cell populations, namely NG_PC_ and HG_PC_.

**Figure 7:**
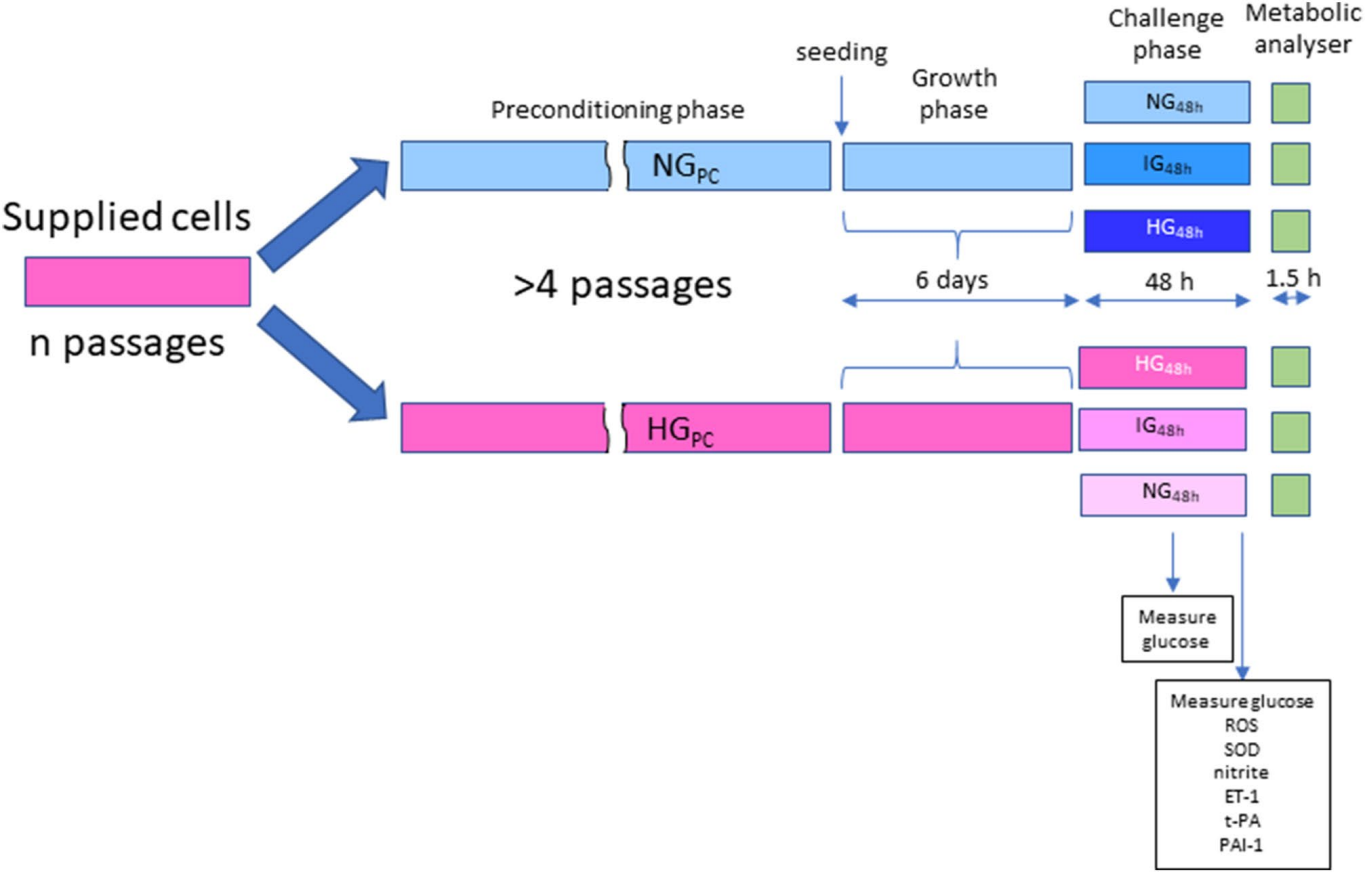
Schematic diagram depicting glucose exposure regimen and outcome measures.

To determine whether pre-conditioning affected cell growth or survival, growth curves were established for both NG_PC_ and HG_PC_ cells. HG_PC_ and NG_PC_ cells (1.9 × 10^5^) were seeded into multiple T-25 flasks, with replicate flasks trypsinised and counted using a haemocytometer (Mariennfeld, Germany) every day for 6 days. Concurrently, a daily visual inspection of the cells was made by light microscopy to assess and record cell morphology and adherence. A schematic diagram of the cell culture protocol is illustrated in Fig. 7.

### Measurement of glucose depletion

A OneTouch Verio Flex glucose meter (LifeScan) was used to determine the concentration of glucose in medium added to NG_PC_ and HG_PC_ cells seeded at 2 × 10^4^ cells/well in a 96-well plate and treated separately with either NG, HG or an intermediate glucose concentration of 12.5 mM (IG) for 48 h. Glucose concentration in the media was measured in triplicate at the start and end of the incubation period.

### Metabolic analysis

The cellular bioenergetic phenotype of both NG_PC_ and HG_PC_ cells was determined by using a Seahorse Bioscience XF^e^96 Extracellular Flux Analyzer (Agilent) to measure the oxygen consumption rate (OCR) and the extracellular acidification rate (ECAR) in the mito-stress test (MST; Agilent). Each assay was performed according to the manufacturer’s instructions and all Seahorse-related kits, reagents and consumables were purchased from Agilent unless stated.

Briefly, the MST assay was used to measure cellular basal and then real-time changes in OCR and ECAR following three sequential injections of mitochondrial electron transport chain (ETC) modulators, namely oligomycin (1 µM; complex V inhibitor), carbonyl cyanide-*4*-(trifluoromethoxy) phenylhydrazone (FCCP, 1 µM; an uncoupler of mitochondrial oxidative phosphorylation) and finally a combination of rotenone and antimycin A (both 0.5 µM), to inhibit complex I and complex III respectively. For this assay, both NG_PC_ and HG_PC_ cells were plated into a Seahorse XF96 cell culture 96-well microplate (2 × 10^4^ cells/well) and left for 12 h to adhere at 37 °C, 5% CO_2_ in a humidified atmosphere. Treatment consisted of addition of NG, IG or HG medium to both NG_PC_ and HG_PC_ cells, followed by incubation under standard conditions for 48 h. The treated cells reached full confluence by conclusion of the incubation period.

On the day of the MST assay, Seahorse assay medium was prepared by adding 1 mM pyruvate, 2 mM l-glutamine and 10 mM glucose to Seahorse XF Base Medium, then adjusted to pH 7.4, filtered and warmed to 37 °C. Medium treatments were removed from each well, washed twice and replaced with assay medium prior to incubation at 37 °C for 45 min in a CO_2_-free incubator. All injectable treatments were freshly prepared, loaded into the injection ports and the sample plate loaded into the Seahorse XF^e^96 for analysis. Data was acquired starting from the third measurement of each cycle and analysed using Seahorse Wave Software (Version 2.6.0.31).

At the end of the assay, the protein content of each well was quantified using a Coomassie-BradfordProtein Assay Kit (Merck, Sigma Aldrich) according to the manufacturer’s instructions, to allow for normalisation of data. All cells were lysed with distilled water and repeated freeze–thaw cycles. This protein estimate assay was subsequently used throughout as a surrogate for the density of treated cells where removal of cells for counting was not practical.

### Total reactive oxygen species/reactive nitrogen species (ROS/RNS) assay

The total ROS/RNS and superoxide detection fluorescent assay (Enzo Life Sciences, UK) was used to estimate the ROS/RNS production of HG_PC_ and NG_PC_ cells seeded at 2 × 10^4^ cells/well in a 96-well plate and treated for 48 h with NG, IG or HG medium. Pyocyanin was used as a positive control for induction of ROS (200 µM for 20 min) and all treatments were made in triplicate. The assay was performed according to the kit instructions. The nature of fluorescent probe for ROS is proprietary information not released by the manufacturer; fluorescence was detected at excitation/emission wavelengths of λ = 488/520 nm (green) using a Varioskan plate reader (Thermo-Fisher) with SkanIt Software 2.4.5 (Varioskan Flash). The results were normalized to the protein content of each well using the method described above.

### SOD activity assay

The Superoxide Dismutase Assay kit (Cayman) was used for the detection of total SOD activity of HG_PC_ and NG_PC_ cells seeded at 5.4 × 10^5^ cells/well in a 6-well plate and treated for 48 h with NG, IG or HG medium to the three test-glucose conditions. The assay utilises a tetrazolium salt for the detection of superoxide anion, generated by xanthine oxidase and hypoxanthine. Results are expressed as U/ml, where 1 unit is the amount of enzyme necessary to dismutate 50% of the superoxide produced. The SOD Assay kit reagents were prepared following the manufacturer’s instructions. Following treatment, NG_PC_ and HG_PC_ cells were lysed in cold lysis buffer (20 mM HEPES buffer, pH 7.2, containing 1 mM egtazic acid (EGTA), 210 mM mannitol and 70 mM sucrose) with the aid of a sonicator, and the lysate solution centrifuged at 1500 g (5 min, 4 °C). The absorbance of the supernatant was measured at λ = 450 nm using the Varioskan platereader. After calculating the average absorbance for each sample or standard, and subtracting the background absorbance, the linearised rate (LR) was calculated. Together with the standard curve, LR allowed the calculation of SOD activity using the following equation:

$${\text{SOD }}\left( {{\text{U/mL}}} \right) = \left\{ {\left[ {\left. {\left( {{\text{sample LR - y - intercept}}} \right)/{\text{slope}}} \right) \times \left( {0.23/0.01\;{\text{mL}}} \right)} \right] \times {\text{sample dilution factor}}} \right\}$$

### Nitrite assay

Nitrite accumulation was measured as a marker of NO production using a Sievers 280i nitric oxide analyser (NOA). NG_PC_ and HG_PC_ were seeded in triplicate in a 96-well plate at 2 × 10^4^ cells/well and treated with NG, IG or HG medium for 48 h under standard conditions. The eNOS-inhibitor, *N*^ω^-nitro-l-arginine methyl ester (l-NAME; 100 µM for 24 h) and calcium ionophore A23187 (1 µM for 1 min) were used as negative and positive controls respectively, to confirm the role of eNOS in the nitrite measured. The medium from each triplicate was collected into a single tube and stored at – 80 °C for batch measurement. Test samples were defrosted on ice, diluted 1 in 4 in deionized water and 100 µl injected into the NOA chamber containing 4.5 ml of glacial acetic acid (Fisher Scientific), 100 µl of diluted antifoam B emulsion and 0.5 ml of sodium iodide (25 mg/ml; Fisher Scientific). Data were acquired using Liquid Software (version 2), integrated and then sample nitrite values extrapolated from the standard curve.

### Measurement of endothelin-1 (ET-1), tissue plasminogen activator (t-PA) and plasminogen activator inhibitor-1 (PAI-1)

Secretion of ET-1, t-PA and PA-1 by NG_PC_ and HG_PC_ exposed to NG, IG or HG medium (48 h) was measured by ELISA. For each ELISA, cell supernatants were prepared by seeding cells in triplicate in 96-well plates (2 × 10^4^ cells/well) and exposing them to NG, IG or HG medium for 48 h under standard conditions. Medium from each triplicate was collected into a single tube and stored at − 80 °C for batch measurement. Samples were processed according to the manufacturer’s instructions for ET-1 (Abcam), t-PA antigen (Zymutest; Hyphen BioMed) and PAI-1 antigen (Zymutest; Hyphen BioMed), with absorbance measured at 450 nm using a Varioskan platereader with with SkanIt Software 2.4.5 (Varioskan Flash, Thermo).

### Statistics

All data are expressed as mean ± SD unless otherwise stated; individual points on each graph indicate independent experiments. Data were analysed using appropriate parametric and non-parametric tests, as defined by normality (Kolmorov–Smirnov test) and equal variance testing. Significant difference between means was accepted at P < 0.05. Statistical tests were performed using GraphPad Prism version 6.00 software (GraphPad Software, San Diego, CA). Details of tests used are included in each figure legend; data were treated as unpaired in all data sets.

### Ethics approval and consent to participate

This was exclusively an in vitro project so consent to participate was not required. The project had internal university ethics approval to proceed.

## Supplementary information

Supplementary Figures.

## Data Availability

The datasets generated during and/or analysed during the current study are available from the corresponding author on reasonable request.

## Abbreviations

ATP: Adenosine triphosphate
CVD: Cardiovascular disease
COX: Cyclo-oxygenase
DMEM: Dulbecco’s Modified Eagle’s Medium
EGTA: Egtazic acid
ETC: Electron transport chain
eNOS: Endothelial nitric oxide synthase
ET-1: Endothelin-1
FCCP: Carbonyl cyanide-*4*-(trifluoromethoxy) phenylhydrazone
HG: High glucose
IG: Intermediate glucose
MST: Mitochondrial stress test
l-NAME: *N*^ω^-Nitro-l-arginine methyl ester
NOX: NADPH oxidase
NO: Nitric oxide
NMOCR: Non-mitochondrial oxygen consumption rate
NG: Normal glucose
OGT: *O*-GlcNAc transferase
OCR: Oxygen consumption rate
PAI-1: Plasminogen activator inhibitor-1
PC: Pre-condition
PTMs: Post-translational modifications
t-PA: Tissue plasminogen activator
ROS: Reactive oxygen species
RNS: Reactive nitrogen species
SOD: Superoxide dismutase
