# Supplementary material for: The impact of glucose exposure on bioenergetics and function in a cultured endothelial cell model and the implications for cardiovascular health in diabetes
Source: Sci Rep. 2020 Nov 11;10:19547. doi: 10.1038/s41598-020-76505-4 (PMC7659000; doi:10.1038/s41598-020-76505-4)
Supplement: Supplementary file 1 — Supplementary Figures. [file 41598_2020_76505_MOESM1_ESM.pdf]

The impact of glucose exposure on bioenergetics and function in a cultured endothelial cell model and implications for cardiovascular health in diabetes

Maria Luisa Fiorello, Andrew T Treweeke, David P Macfarlane, Ian L Megson

Supplementary Fig S1

A

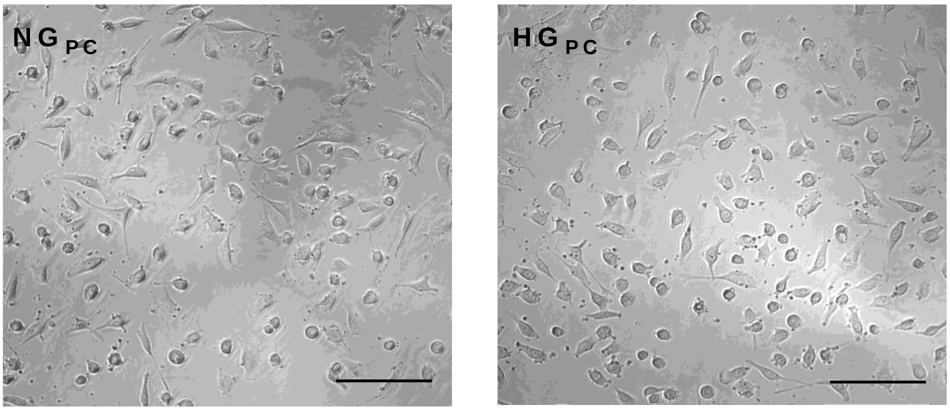

B

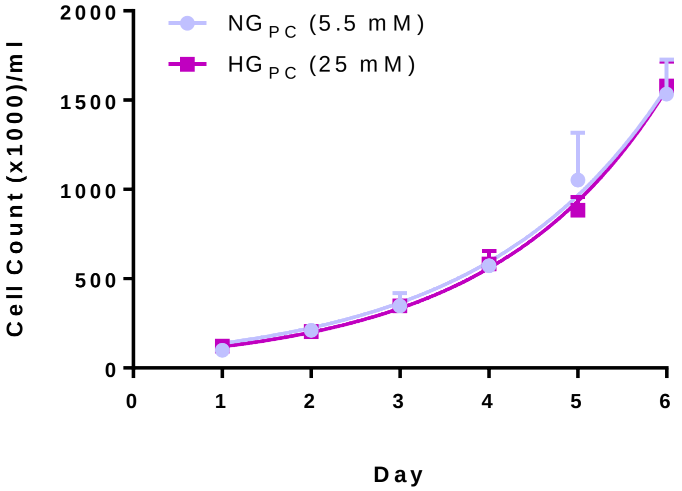

Supplementary Fig 1. *Cell morphology (A) and growth rate (B) in cells cultured through >5 passages under normoglycaemic (5.5 mM starting concentration; NG_{PC}) or hyperglycaemic (25 mM starting concentration; HG_{PC}) conditions. There was no apparent change in morphology or growth rate ($P>0.05$; 2-factor ANOVA) induced by NG_{PC}. Scale bar: 150 μm. Cells used for treatment experiments were passaged at ~90% confluence and reached full confluence by conclusion of the incubation period.*

Supplementary Fig S2

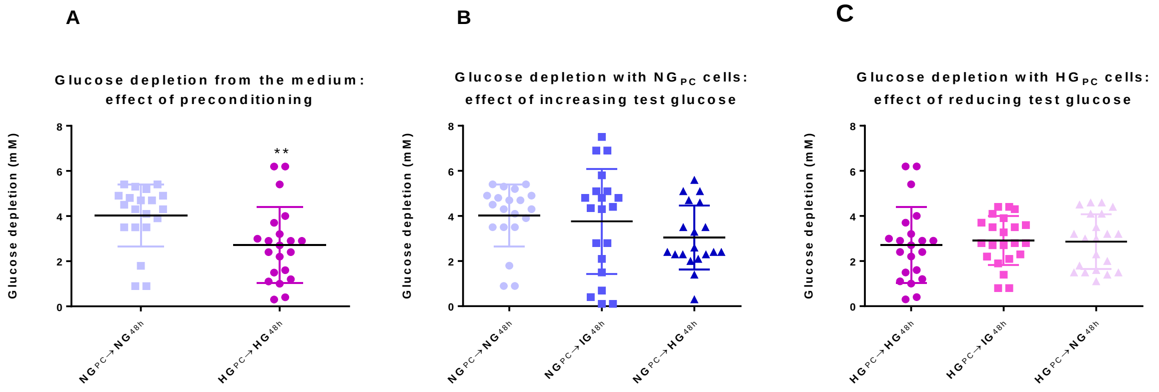

Supplementary Fig S2. *(A) Effect of glucose preconditioning on glucose depletion from medium over 24 h (***P<0.001; Mann Whitney U test). (B) Effect of 48 h exposure of increasing glucose concentrations to NG_{PC} cells on glucose depletion from the medium (P>0.05; Kruskal Wallis). (C) Effect of 48 h exposure of decreasing glucose concentrations to HG_{PC} cells on glucose depletion from the medium (P>0.05; Kruskal Wallis).*

Supplementary Fig S3

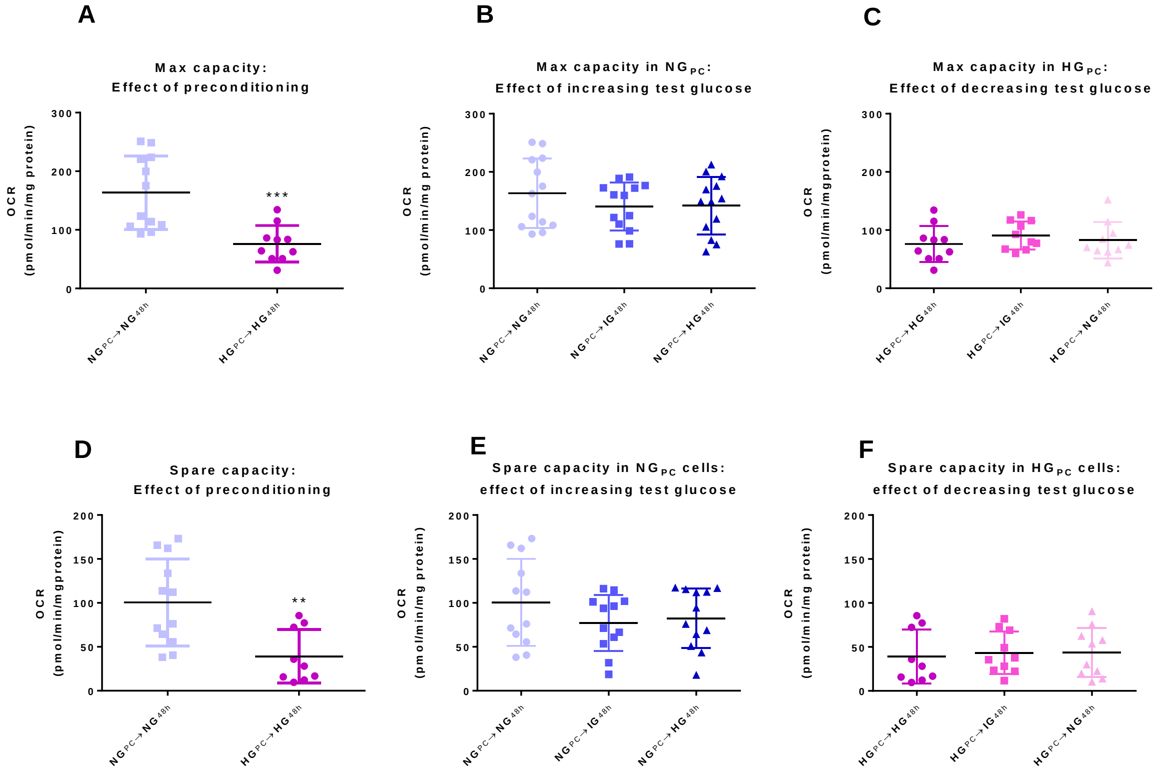

Supplementary Fig S3. *Effect of glucose preconditioning on maximum capacity (A) and spare capacity (D) (***P<0.001; **P<0.01, Mann Whitney U test). Effect of 48 h exposure of increasing glucose concentrations to NG_{PC} cells on maximum capacity (B) and spare capacity (E) (P>0.05; Kruskal-Wallis). Effect of 48 h exposure of decreasing glucose concentrations to HG_{PC} cells on maximum capacity (C) and spare capacity (F) (P>0.05; Kruskal-Wallis).*
